# Comprehensive profiling of bioactive compounds in germinated black soybeans via UHPLC-ESI-QTOF-MS/MS and their anti-Alzheimer’s activity

**DOI:** 10.1371/journal.pone.0263274

**Published:** 2022-01-28

**Authors:** Umair Shabbir, Akanksha Tyagi, Hun Ju Ham, Deog-Hwan Oh

**Affiliations:** 1 Department of Food Science and Biotechnology, College of Agriculture and Life Sciences, Kangwon National University, Chuncheon, The Republic of Korea; 2 Department of Biological Environment, College of Agriculture and Life Sciences, Kangwon National University, Chuncheon, The Republic of Korea; Amity University Kolkata, INDIA

## Abstract

Black soybeans contain several bioactive compounds and commonly consumed due to their health-related activities but rarely cultivated as edible sprouts. The present study investigated the changes that occurred during germination in two new genotypes black soybeans. Raw and germinated seeds were tested against in vitro Alzheimer’s disease (AD) biomarkers, including oxidative stress, inflammatory factors and cholinesterase enzymes as well as γ-aminobutyric acid (GABA) levels. Sprouts significantly inhibited the cholinesterase enzymes and inflammatory factors (protein denaturation, proteinase and lipoxygenase) than seeds. An increase in phenolic, flavonoid and GABA (10-folds) content and antioxidant capacity (ABTS, DPPH, and FRAP) was observed in germinated seeds. However, anthocyanin content was decreased in sprouts. UHPLC-ESI-QTOF-MS^2^ metabolites profiling approach identified 22 compounds including amino acids, peptides, fatty acids, and polyphenols. Among identified compounds, daidzein, genistein, gallic acid, spermidine, L-asparagine, and L-lysine exhibited the highest increase after germination. The current study reveals that germination of black soybeans have promising potential to inhibit/prevent AD and can be used to develop functional foods.

## 1. Introduction

Alzheimer’s disease (AD) is a neurodegenerative disease that is characterized by the gradual onset and progressive deterioration in cognitive function [1]. It contributes about 60–70% cases of dementia and is considered as the 7^th^ prominent cause of deaths with one of the primary roots of dependency and disability among the elderly population [2]. AD not only poses psychological, physical, and economical problems but also have a great impact on society and families [3]. The formation of oxidative stress, inflammatory signaling, neurofibrillary tangles, amyloid-beta (Aβ) peptides in the nerve cells, and variations in neurotransmitter signaling systems such as acetylcholine (ACh), and γ-aminobutyric acid (GABA) are leading to the AD [4]. GABA is an essential amino acid that serves as an inhibitory neurotransmitter for brain and plays role in information processing, neuronal development and cognition. Studies are suggesting that aggregation of Aβ peptides in the hippocampus interfere with GABA inhibitory interneuron function and promote memory impairment [5]. Further, defects in the cholinergic system can reduce the ACh levels in the brain due to the loss of cholinergic neurons and neurotransfomation. Based on the cholinergic hypothesis, the inhibition of acetylcholinesterase (AChE) and butyrylcholinesterase (BChE) and increment in ACh levels can improve the memory and cognitive functions in AD patients [6, 7]. Moreover, oxidative stress and inflammation are closely associated and can activate microglia, astrocyte, cytokines and chemokines to disrupt the blood-brain barriers and contribute to neurodegeneration [8]. Thus, the elevation of GABA and ACh in brain and the inhibition of inflammation, oxidative stress, AChE and BChE can be promising strategies to treat AD.

Black Soybean (*Glycine max* L.) is one of the most nutritious crops and commonly consumed due to its therapeutic activities. It is rich in phytochemicals such as anthocyanins, isoflavone, flavan-3-ols, phenolic compounds that possess antioxidant, anti-inflammatory, anticarcinogenic, and antimutagenicity activities [9]. Interestingly, the health beneficial properties not only depend on the phenolic content but also the concentration and composition of individual compounds. Black soybean is classified as seoritae cultivars that have greenish cotyledon and have higher phenolic compounds than white and yellow seed-coated cultivars [10]. Germination of black soybean can activate the seed metabolites and induce hydrolysis of high-molecular-weight polymers via the activity of enzymes. It triggers the functional activity by enhancing the phytochemicals, including polyphenolic, and non-polyphenolic compounds, amino acids, GABA, and vitamins [11].

Metabolomics is an emerging field to screen the whole spectrum of metabolites in a biological specimen and untargeted metabolomics approaches are helpful to compare the metabolite profiles of different sample extracts. Therefore, the present study investigated the alteration caused by the germination of the bioactive compounds using UHPLC-ESI-QTOF-MS^2^ of two new genotypes of black soybeans grown in the Republic of Korea. Moreover, to analyze the effect of germination on the bioactive compounds and their health-related activities and to examine in vitro anti-Alzheimer’s (antioxidative, inhibition of inflammatory factors, anti-cholinesterase, GABA level) activity of these seeds. To our knowledge, UHPLC-ESI-QTOF-MS^2^ metabolite profiling technique has not been implied yet to characterize the entire metabolic profile of black soybeans and their sprouts. Further, this is the first study that compares the antioxidative, anti-cholinesterase activity, inhibition of inflammatory factors, and GABA levels of raw and germinated black soybeans. Thus, this study can be helpful in the development of functional foods using germinated black soybean genotypes to prevent/inhibit neurodegenerative and other chronic disoders.

## 2. Materials and methods

Black soybean genotypes used in this study were provided by the National Institute of Crop Science, Rural Development Administration-South Korea. Soybean samples were named BS1 (Se-Um) and BS2 (Miryang 365) (S1 Fig). Dried sprouts and seeds were ground into a fine powder using the electric mill and sifted through mesh 40 sieve and stored at -20°C.

### 2.1. Reagents and chemicals

Chemicals and reagents such as gallic acid, Folin–Ciocalteau’s reagent, bovine serum albumin, catechin, trypsin, cyanidine 3-o-glucoside chloride, aspirin, perchloric acid, casein, Tris-HCl, lipoxygenase, linoleic acid, dithiobis nitrobenzoic acid (DTNB), acetylthiocholine iodide (ATCI), butyrylcholine iodide (BTCI), galanthamine, 1,1-Diphenyl-2-picrylhydrazyl (DPPH), acetylcholinesterase (AChE) Electric eel (CAS 9000-81-1), 2,2′-azinobis-3-ethylbenzothiazoline-6-sulfonic acid (ABTS), BChE equine serum lyophilized (CAS 9001-08-5), and trolox were procured from Sigma. Ethanol, methanol and all other analytical grade organic solvents were obtained from Daejung Chemicals & Metals Co., Ltd., Korea.

### 2.2. Preparation of germinated black soybean

Both samples (50 g each) were disinfected by immersing in a 0.2% sodium hypochlorite solution with gentle shaking for 10 minutes. Afterwards, seeds were splashed with tap water three times and soaked into water for 8 hours. After draining the water, seeds were cultured in petri dishes accommodating Murashige and Skoog medium (25 mL). These seeds were germinated for 1–5 days at 25± 2°C in a tissue culture incubator (LIB-010M; DAIHAN LABTECH CO., LTD.) under dark (S1 Fig). Following germination, sprouts were dried in a blast drying oven (OF-22GW, JEIO Tech Instrument Co., Ltd., Seoul, Korea) at 55°C for 6 hours. Initial screening of the seeds and sprouts was done on the basis of antioxidant capacity and inhibition of inflammatory factors (S1 Table). Significant improvement was found on the 3^rd^ day of germination in both genotypes compared to other days. Therefore, raw and germinated seeds (3^rd^ day) were used for further analyses.

### 2.3. Sample preparation

Extraction of powdered samples (5 g) was done with ethanol (70%; 1:20 w/v) in an orbital shaker for 1 hour at 50°C. Thereafter samples were centrifuged at 4000×g for 15 minutes and the supernatant was collected while the residues were extracted again (up to 3^rd^ extraction) under the mentioned conditions. Supernatants were combined and concentrated under the vaccum at 40°C. Afterwards, freeze-dried and the lypholized solids were kept at -20°C. The stock solution of samples were reconstituted in 70% ethanol or 10% DMSO for further analysis.

### 2.4. Determination of total phenolic content (TPC)

The TPC of ethanol extracts (1mg/mL) was measured by following the Folin–Ciocalteu colorimetric method with gallic acid (standard) as documented by Guo et al. [12] with minor variations. Shortly, a 100-μL aliquot of each extract, methanol blank 95% (v/v) or standard was combined with Folin-Ciocalteu reagent (200 μL) using vortex followed by incubation for 2 hours. Then, 800 μL of 700 mM Na_2_CO_3_ was pipetted and absorbance was measured using SpectraMax i3 plate reader (Molecular Devices Korea, LLC) at 765 nm. The concentration of TPC was represented as mg gallic acid Equiv./ g dry weight (DW).

### 2.5. Determination of total anthocyanin content (TAC)

The anthocyanins were determined by following the method of Lee et al. [13] with some amendments. In short, 0.1 g of each freeze-dried sample was dissolved in 10 mL of 60% ethanol containing 1% citric acid and thoroughly mixed with vortex and absorbance was measured at 535 nm using the spectrophotometer (Evolution 201, Thermo, Waltham, MA, USA). Cyanidine 3-O-Glucoside chloride (C3G) was used as a standard to create the curve and TAC was measured as mg C3G Equiv./ g DW.

### 2.6. Determination of total flavonoid content (TFC)

The TFC was conducted as reported by Ofosu et al. [14] with slight changes. In short, 250 μL ethanol extracts (1mg/mL), blank or standard was added and mixed with NaNO_2_ (75μL; 50 g/L) and 1 mL distilled water. The reaction mixture was incubated (5 min) and AlCl3 (75 μL; 100 g/L) was mixed and the mixture was settled for 6 min. Afterwards, 600 μL of distilled water followed by 500 μL of 1 M NaOH was mixed. The absorbance was taken at 510 nm after shaking (20 sec) in the SpectraMax i3 plate reader. The concentration of TFC was examined from the standard (catechin) and presented as mg catechin Equiv./ g DW.

### 2.7. Determination of antioxidant activity

The antioxidant capacity of ethanol extracts (1mg/mL) was determined by examining their ferric reducing antioxidant power (FRAP assay) through the method of Zeng et al. [15] and free radical scavenging activities (DPPH and ABTS assay) by Xiang et al. [16] with minor changes in the protocols. Concisely, 2 mL of FRAP reagent was mixed with sample (0.2 mL) and incubated for 10 min at 37°C, the absorbance was measured at 593 nm. Moreover, freshly prepared 100 μL of 500 μM DPPH (in methanol) was pipetted with 100 μL (1 mg/mL concentration) of sample extract or Trolox in microplate and incubated for 30 min in the dark to determine the DPPH activity. The absorbance was read at 517 nm. The ABTS assay was determined by mixing the 100 μL of sample extract with 1 mL of ABTS solution and the absorbance was measured at 734 nm. The results of all antioxidant assays were presented as mg Trolox equiv./ g, DW.

### 2.8. Detection of gamma-aminobutyric acid (GABA)

The GABA content of the ethanolic extract was measured via following the method of Liu et al. [17] (slightly modified the procedure). The HPLC was used for GABA detection having the Poroshell HPH-C18 column (4.6 × 100 mm, 2.7 μm) using pre-column derivatization with dansyl chloride. The derivatization method was as follows: extract samples (1mg/mL) and 0.2 mL of GABA standard solution (different concentration: 1, 0.5, 0.25, 0.12, 0.06 mg/mL) were mixed with 0.2 mL dansyl chloride solution in a 1 mL brown volumetric flask and shaken. Then samples and standards were derived at 55°C in the water bath (SH-Wb-11GDN, SH Scientific, Korea) for 50 minutes under dark followed by cooling at room temperature and diluted to a volume of 1.0 mL with methanol and filtered with 0.45 μm membrane. The pH of the samples was maintained at 8±0.5 by adding 0.1 mol/L NaHCO₃. The GABA content was measured through the standard curve of GABA and results were expressed as mg/mL.

Following HPLC conditions were used: HPLC (Agilent Technologies, Baudrats, Germany) equipped with an Infinity Lab Poroshell HPH-C18 column (4.6 × 100 mm, 2.7 μm) fitted with a guard column (Agilent Technologies, CA, USA); a column temperature of 30°C; a flow rate of 1 mL/min; an amount of injection of approximately 2 μL with mobile phase A, methanol and mobile phase B, water.

### 2.9. In-vitro inhibition of inflammatory factors

The effect of black soybean extracts on inflammatory factors (in vitro) were assessed by examining the protein denaturation, anti-proteinase and anti-lipase activities as described Gunathilake et al. [18] with some modifications. The freeze-dried sample extracts were serially diluted (25 to 500 μg/mL) in dimethyl sulfoxide (DMSO). DMSO was used as a negative control and standard anti-inflammatory drug, Aspirin (100 μg/mL) was used as a positive control.

#### 2.9.1. Inhibition of protein denaturation

A 5 mL reaction mixture was prepared containing 0.02 mL (1mg/mL) extract or standard (aspirin), 4.78 mL phosphate-buffered saline (pH 6.3), and 0.2 mL bovine albumin (1%) was mixed thoroughly and the pH of the mixture was adjusted at 6.3. After, incubated in the water bath at 37°C for 15 min. Afterwards, the reaction mixture was heated for 4 min at 80°C. The turbidity was measured at 660 nm using the UV-Visible spectrophotometer. The inhibition percentage of protein was measured using the equation (below) and data were expressed as IC_50_ (concentration of test extracts required for 50% inhibition):

Percentageinhibition=100×Ac–As/Ac

Where Ac is absorption of the negative control and As is the absorption of test sample.

#### 2.9.2. Inhibition of proteinase

Trypsin (0.06 mg) was mixed with 1 mL of test sample (1mg/mL) and 1 mL of 20 mM Tris-HCl buffer (pH 7.4) and incubated for 5 min at 37°C. After, 1 mL of 0.8% (*w/v*) casein was added into the mixture and further settled for 20 minutes. 2 mL perchloric acid (70%) was added to stop the reaction and cloudy suspension was centrifuged at 6000 rpm for 10 min and the supernatant was used for absorbance at 210 nm. The percentage inhibition was measured using the same formula as used for the inhibition of protein denaturation (mentioned above) and data were expressed as IC_50_.

#### 2.9.3. Lipoxygenase inhibition activity

Lipoxygenase (10 μL, 8000 U/mL concentration) was mixed with 1 mL sodium borate buffer (0.1 M, pH 8.8) and 10 μL of extract (1mg/mL) and incubated for 5 minutes at room temperature. Linoleic acid substrate (10 μL; 10 mmol) was used to initiate the reaction and absorbance was measure at 234 nm. Percentage inhibition was measured with the same formula as used for the inhibition of protein denaturation and data were expressed as IC_50_.

### 2.10. Anticholinesterase assays

Inhibition of cholinesterase enzymes (AChE and BChE) was measured via following the protocol of Ellman et al. [19] by slightly modifying the procedure. 96-well plates were used to prepare the reaction mixture. An anticholinesterase drug (Galantamine) was used as a standard. 20 μL solution of AChE (5.32 × 10 ^−3^ U) or BChE (6.85 × 10 ^−3^ U) was incubated with different concentrations (100–500 μg/mL) of sample solutions (dissolved in 10% DMSO) in 150 μL of 100 mM sodium phosphate buffer (pH 8.0) for 45 minutes at room temperature. Then, 0.5 mM DTNB (10 μL) was added and ACTI (0.71 mM) or BTCI (0.2 mM) was mixed to start the reaction. The hydrolysis of these substrates was observed by the formation of yellow-colored 5-thio-2-nitrobenzoate anion at 412 nm using the spectrophotometer.


$$\%\;Inhibition=\frac{control\;absorbance-sample\;absorbance}{control\;absorbance}\times 100$$

AChE and BChE assay results were presented in IC_50_.

### 2.11. Identification of metabolites

The bioactive metabolites were analyzed using the UHPLC-ESI-QTOF-MS^2^ (SCIEX Exion LC AD system, Framingham, MA, USA) by following the protocol previously used in our laboratory by Ofosu et al. [14]. Both positive (ESI+) and negative (ESI−) ion modes were used to conduct the mass spectrometric analysis. In short, the UHPLC SCIEX Exion LC AD system was assembled with different components, including photodiode array detector, a controller, and AD autosampler. The analytical column consisted of a 100 mm×3 mm Accucore C18 column. Then, 10 μL of ethanol extracts (1mg/ml) was injected and eluted into the column with a binary mobile phase comprising of several components denoted as A (water containing 0.1% formic acid) and B (methanol). A flow rate (0.4 mL/min) with a linear gradient programmed for 25 min as demonstrated: 9–14% B (0–3.81 min), 14–15% B (3.81–4.85 min), 15% B (4.85–5.89 min), 15–17% B (5.89–8.32 min), 17–19% B (8.32–9.71 min), 19% B (9.71–10.40 min), 19–26% B (10.40–12.48 min), 26–28% B (12.48–13.17 min), 28–35% B (13.17–14.21 min), 35–40% B (14.21–15.95 min), 40–48% B (15.95–16.64 min), 48–53% B (16.64–18.37 min), 53–70% B (18.37–22.53 min), 70–9% B (22.53–22.88 min), and 9% B (22.88–25.00 min). The scanning time was about 1 second under these conditions. The components in the raw and germinated samples were identified by using a metabolomics workbench.

### 2.12. Statistical analysis

The obtained data were statistically analyzed using the Graphpad Prism 8.0 (GraphPad Software, San Diego, USA). One-way analysis of variance (ANOVA) and Tukey’s test at p < 0.05 significance level was used to find the difference of mean values of the black soybean extract activities. All results were represented as mean ± standard deviation (SD). The heat map and principal component analysis (PCA) were performed using the ClusVis (https://biit.cs.ut.ee/clustvis/) (accessed on 02 October 2021).

## 3. Results

### 3.1. Identification of metabolites

Plant metabolites have biological activities and known as phytotherapeutics to prevent a variety of acute and chronic diseases. The present study was conducted to analyze the effect of sprouting on the bioactive metabolites of two genotypes of black soybean. The metabolite profile was inspected using the UHPLC-Q-TOF-MS/MS-based metabolites profiling technique. The metabolites were identified based on antioxidant, anti-inflammatory, and anticholinesterase activities. The strength of the positive and negative ion models was compared to identify the metabolites and obtained spectral data was used to identify the metabolites tentatively in the germinated and non-germinated seeds (shown in Table 1). Although both of the ion models presented almost similar metabolites but positive ion model identified more metabolites. Identification and characterization of metabolites were achieved by comparing the present study results with mass spectral literature evidence and cross-referencing it with Metlin and metabolomics workbench. Total 22 metabolites were identified in the sprouts and higher metabolites were identified in the sprouts as compared to the seeds. Enhanced content of identified metabolites (amino acids, phenolic compounds, fatty acids, and peptides) was observed in sprouts. Among polyphenols genistein, naringenin, p-coumaric acid, gallic acid, daidzein, and (+)-catechin were predominantly increased especially in BS2 sprouts. Some peptides such as phenylalanylleucine, Leu-Leu-Gly, Asn-Phe, and Leu-Phe were also identified in the extracts. However, phenylalanylleucine and Asn-Phe were only present in sprouts. Essential amino acids, including L-lysine, L-phenylalanine, L-arginine, L-tryptophan, L-histidine, and L-valine were identified and the germination process positively increased their content in the sprouts especially in the BS2 variety.

**Table 1:** Biochemical compounds identified from by UHPLC−Q-TOF-MS/MS from raw and sprouted black soybeans, RT: Retention time.

S.no.	Extract	RT (min)	Peak area	Precursor Mass	Found at Mass	Molecular formula	Compound identified
**1**	BS1 seed	0.81	1.23×10^5^	132.059	133.060	C_4_H_8_N_2_O_3_	L-Asparagine
	BS1 sprout	0.79	1.50×10^6^	133.059	131.056		
	BS2 seed	0.79	1.41×10^5^	132.059	133.060		
	BS2 sprout	0.81	1.60×10^6^	132.059	131.054		
**2**	BS1 seed	Nd	Nd	Nd	Nd	C_6_H_14_N_2_O_2_	L-Lysine
	BS1 sprout	0.67	1.80×10^5^	146.106	147.112		
	BS2 seed	Nd	Nd	Nd	Nd		
	BS2 sprout	0.67	1.61×10^5^	146.106	147.113		
**3**	BS1 seed	8.91	5.26×10^5^	165.079	166.0865	C_9_H_11_NO_2_	L-Phenylalanine
	BS1 sprout	2.94	8.50×10^5^	165.079	166.0864		
	BS2 seed	8.89	6.74×10^5^	165.079	166.0864		
	BS2 sprout	2.93	9.44×10^5^	165.079	166.0864		
**4**	BS1 seed	0.72	3.20×10^6^	174.111	175.118	C_6_H_14_N_4_O_2_	L-Arginine
	BS1 sprout	0.70	7.00×10^6^	174.111	175.118		
	BS2 seed	0.70	3.27×10^6^	174.111	175.118		
	BS2 sprout	0.72	6.40×10^6^	174.111	175.118		
**5**	BS1 seed	5.87	3.38×10^5^	204.097	205.097	C_11_H_12_N_2_O_2_	L-Tryptophan
	BS1 sprout	5.89	6.50×10^5^	204.090	205.097		
	BS2 seed	5.85	3.91×10^5^	204.090	205.097		
	BS2 sprout	5.85	7.60×10^5^	204.090	205.097		
**6**	BS1 seed	0.71	3.15×10^5^	155.069	155.15	C_6_H_9_N_3_O_2_	L-Histidine
	BS1 sprout	0.71	7.09×10^5^	155.069	155.15		
	BS2 seed	0.72	3.30×10^5^	155.069	155.15		
	BS2 sprout	0.71	7.50×10^5^	155.069	155.14		
**7**	BS1 seed	1.49	3.61×10^5^	117.078	117.151	C_5_H_11_NO_2_	L-Valine
	BS1 sprout	1.50	7.46×10^5^	117.078	117.151		
	BS2 seed	1.51	3.75×10^5^	117.078	117.151		
	BS2 sprout	1.49	7.59×10^5^	117.078	117.151		
**8**	BS1 seed	0.85	3.47×10^5^	272.068	272.251	C_15_H_12_O_5_	Naringenin
	BS1 sprout	0.85	5.40×10^5^	272.068	272.251		
	BS2 seed	0.85	3.70×10^5^	272.068	272.251		
	BS2 sprout	0.87	7.84×10^5^	272.068	272.251		
**9**	BS1 seed	15.21	3.76×10^5^	270.022	270.311	C_15_H_10_O_5_	Genistein
	BS1 sprout	15.20	7.51×10^5^	270.022	270.310		
	BS2 seed	15.20	4.74×10^5^	270.022	270.311		
	BS2 sprout	15.20	9.67×10^5^	270.022	270.310		
**10**	BS1 seed	1.22	1.69×10^5^	164.047	165.054	C_9_H_8_O_3_	p-Coumaric acid
	BS1 sprout	1.23	5.91×10^5^	164.047	165.054		
	BS2 seed	1.22	2.80×10^5^	164.047	165.054		
	BS2 sprout	1.24	8.86×10^5^	164.047	165.053		
**11**	BS1 seed	0.78	4.18×10^5^	484.077	484.079	C_21_H_21_ClO_11_	Cyanidin 3-glucoside
	BS1 sprout	0.78	1.10×10^3^	484.077	484.081		
	BS2 seed	0.77	4.09×10^5^	484.077	484.079		
	BS2 sprout	0.78	1.41×10^3^	484.076	484.080		
**12**	BS1 seed	1.67	3.36×10^5^	170.023	170.124	C_7_H_6_O_5_	Gallic acid
	BS1 sprout	1.66	1.46×10^6^	170.023	170.125		
	BS2 seed	1.67	3.12×10^5^	170.023	170.124		
	BS2 sprout	1.67	1.78×10^6^	170.023	170.125		
**13**	BS1 seed	16.35	1.20×10^5^	254.058	255.065	C_15_H_10_O_4_	Daidzein
	BS1 sprout	16.34	5.80×10^6^	254.058	255.065		
	BS2 seed	16.34	1.73×10^5^	254.058	255.065		
	BS2 sprout	16.35	6.20×10^6^	254.058	255.065		
**14**	BS1 seed	14.98	3.74×10^5^	290.078	290.18	C_15_H_14_O_6_	(+)-Catechin
	BS1 sprout	15.01	7.36×10^5^	290.078	290.17		
	BS2 seed	15.08	3.38×10^5^	290.078	290.17		
	BS2 sprout	14.99	6.93×10^5^	290.078	290.15		
**15**	BS1 seed	0.99	5.18×10^5^	145.157	145.251	C_7_H_19_N_3_	Spermidine
	BS1 sprout	0.98	3.59×10^6^	145.157	145.251		
	BS2 seed	0.99	5.61×10^5^	145.157	145.250		
	BS2 sprout	0.97	4.73×10^6^	145.157	145.250		
**16**	BS1 seed	Nd	Nd	Nd	Nd	C_15_H_22_N_2_O_3_	Phenylalanylleucine
	BS1 sprout	2.01	1.89×10^5^	263.139	263.141		
	BS2 seed	Nd	Nd	Nd	Nd		
	BS2 sprout	2.02	1.45×10^5^	263.139	263.141		
**17**	BS1 seed	4.05	5.42×10^4^	302.207	302.206	C_14_H_27_N_3_O_4_	Leu-Leu-Gly
	BS1 sprout	4.09	4.84×10^5^	302.207	302.206		
	BS2 seed	4.07	1.23×10^5^	302.207	302.206		
	BS2 sprout	4.08	5.98×10^5^	302.207	302.206		
**18**	BS1 seed	Nd	Nd	Nd	Nd	C_13_H_17_N_3_O_4_	Asn-Phe
	BS1 sprout	15.90	4.67×10^5^	280.129	280.129		
	BS2 seed	Nd	Nd	Nd	Nd		
	BS2 sprout	15.91	3.88×10^5^	280.129	280.129		
**19**	BS1 seed	11.34	2.61×10^5^	265.154	265.154	C_14_H_18_N_2_O_3_	Leu-Phe
	BS1 sprout	11.32	4.45×10^5^	265.154	265.154		
	BS2 seed	11.33	1.87×10^5^	265.154	265.154		
	BS2 sprout	11.34	4.23×10^5^	265.154	265.153		
**20**	BS1 seed	Nd	Nd	Nd	Nd	C_18_H_34_O_2_	Oleic acid
	BS1 sprout	4.49	4.00×10^3^	282.256	281.248		
	BS2 seed	Nd	Nd	Nd	Nd		
	BS2 sprout	7.62	1.80×10^3^	282.256	281.248		
**21**	BS1 seed	0.15	4.76×10^4^	180.240	279.233	C_18_H_32_O_2_	Linoleic acid
	BS1 sprout	0.14	5.90×10^4^	180.240	279.233		
	BS2 seed	0.15	5.88×10^4^	180.240	279.232		
	BS2 sprout	0.15	6.35×10^4^	180.240	279.232		
**22**	BS1 seed	1.13	5.34×10^5^	278.224	278.435	C_18_H_30_O_2_	α-Linolenic acid
	BS1 sprout	1.12	5.88×10^5^	278.224	278.435		
	BS2 seed	1.12	5.76×10^5^	278.224	278.434		
	BS2 sprout	1.20	6.01×10^5^	278.224	278.433		

#### 3.1.1. Heat map and PCA

**Fig 1:**
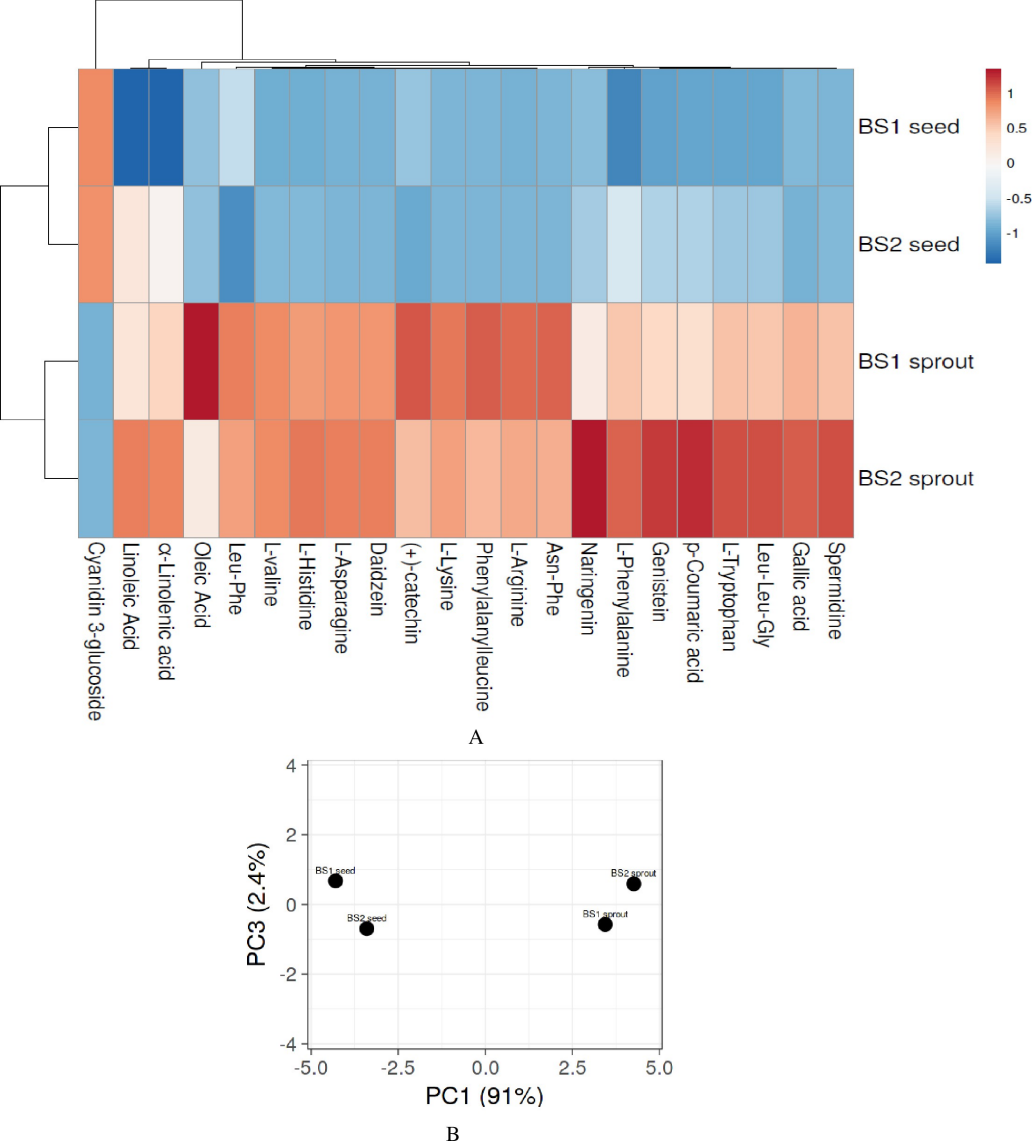
Identified bioactive compounds in black soybean seeds and sprouts. **A**) Heat map plot showing the levels of identified compounds based on colors from red to blue, expressing the level of compounds in decreasing order. **B**) Principle component analysis (PCA) by comparing PC1 with PC3.

A heat map was used to show the alteration in the metabolites for clustering phenolic compounds based on their concentrations (Fig 1A). The blue color indicates compounds with low concentration while the red color shows the high concentration. The PCA was used to investigate the discrimination between the extracts based on metabolic profiles (Fig 1B). The raw BS1 and BS2 were found to be correlated with germinated BS1 and BS2, respectively after comparing PC1 with PC3. The PCA illustrated the same trends as observed in the heat map analysis.

### 3.2. Anticholinesterase activity

**Fig 2:**
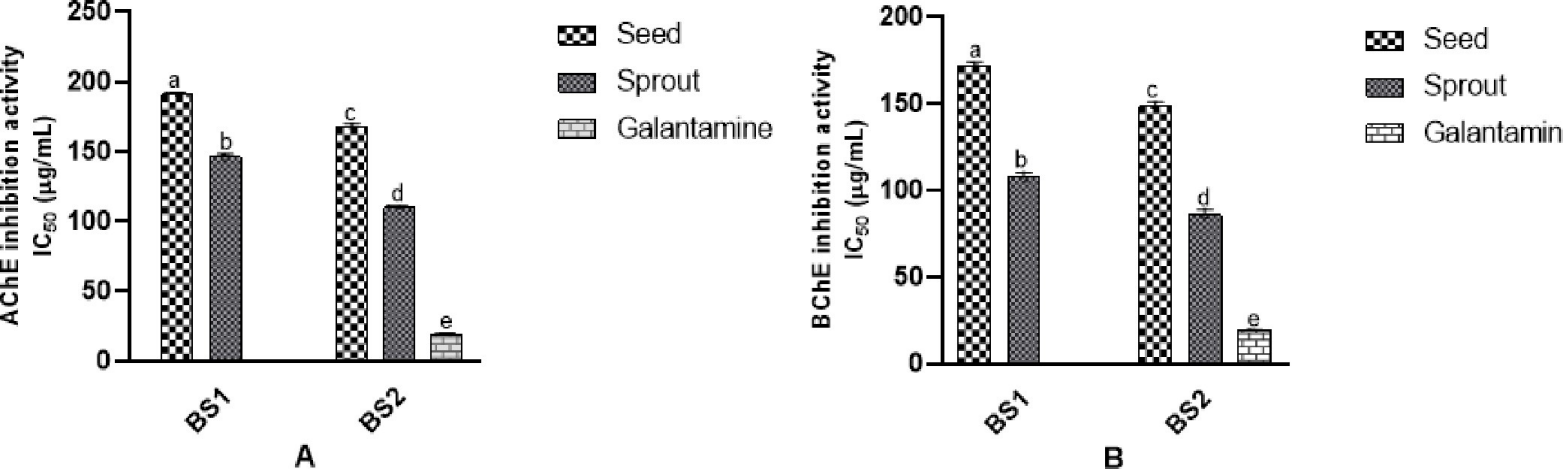
In-vitro anticholinesterase activity of black soybean extracts (1mg/mL). **A**) AChE inhibition activity IC_50_ (μg/mL). **B**) BChE inhibition activity IC_50_ (μg/mL). Values represent triplicate readings and different superscripts denote significant differences (*p* < 0.05).

The ability of sample extracts to inhibit AChE and BChE activities was investigated (in vitro) and results (IC_50_) are expressed in Fig 2A and 2B, respectively. The highest inhibition against AChE was shown by BS2 sprouts (IC_50_ value = 109.64±2.38 μg/mL) and the significant improvement was noticed than the BS2 seeds (IC_50_ value = 167.40±3.16 μg/mL). Moreover, a significant difference was also observed in germinated BS1 (IC_50_ value = 146.37±2.87 μg/mL) than seeds (IC_50_ value = 190.75±1.99 μg/mL). The positive control (Galantamine) inhibited 19.23±0.85 μg/mL (IC_50_).

In the case of BChE, more significant inhibition was observed than AChE. Results (Fig 2B) indicated that the inhibitory capacity of seeds was significantly increased after germination of both varieties. Activity of BS1 (171.79±5.51 μg/mL) and BS2 (148.32±4.91 μg/mL) seeds enhanced in the sprouts of BS1 (107.78±3.64 μg/mL) and BS2 (85.97±5.48 μg/mL), when compared with Galantamine (19.42±0.93 μg/mL). It can be seen from the results that germination of black soybean can enhance the cholinergic neurotransmission by inhibiting the AChE and BChE activities.

### 3.3. Detection of GABA content

**Fig 3:**
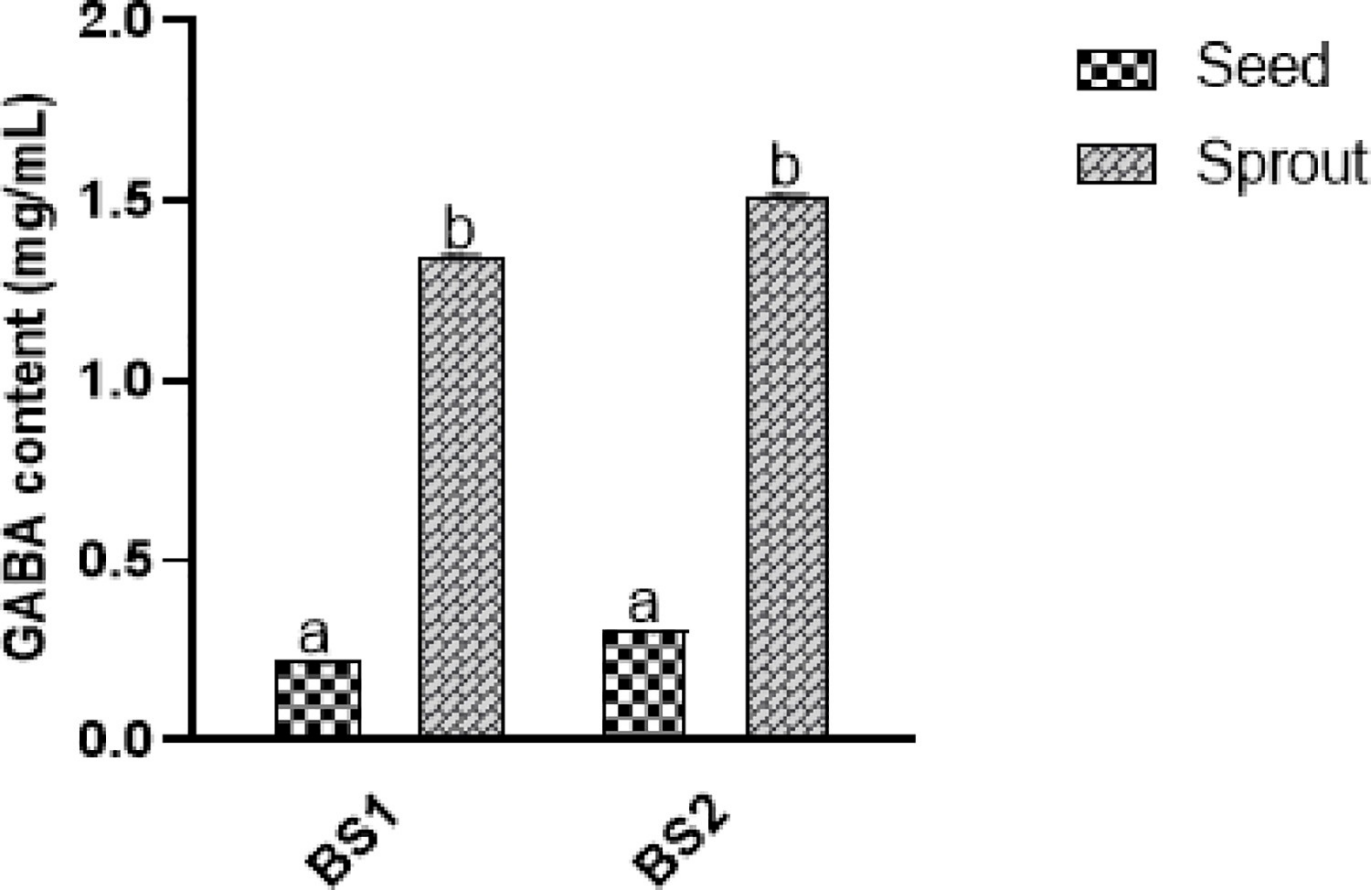
GABA content (mg/mL) in raw and germinated black soybeans. Results were expressed as mean ± SD of triplicate analysis. Data marked with different letters are significantly (*p < 0*.*05*) different.

GABA is a ubiquitous non-protein amino acid and a major inhibitory neurotransmitter that regulates the neuronal excitatory/inhibitory balance. It also plays role in the regulation of blood pressure and heart rate and can stimulate the synthesis of insulin to prevent diabetes. The content of GABA in seeds and sprouts of soybean are illustrated in Fig 3. The GABA content was 0.216 mg/mL and 0.302 mg/mL in BS1 and BS2 seeds while it was 1.343 mg/mL and 1.506 mg/mL in BS1 and BS2 sprouts, respectively. Both germinated soybeans showed about a 10-fold increment in the GABA content.

### 3.4. Inhibition of inflammatory factors

#### 3.4.1. Inhibition of protein denaturation

**Fig 4:**
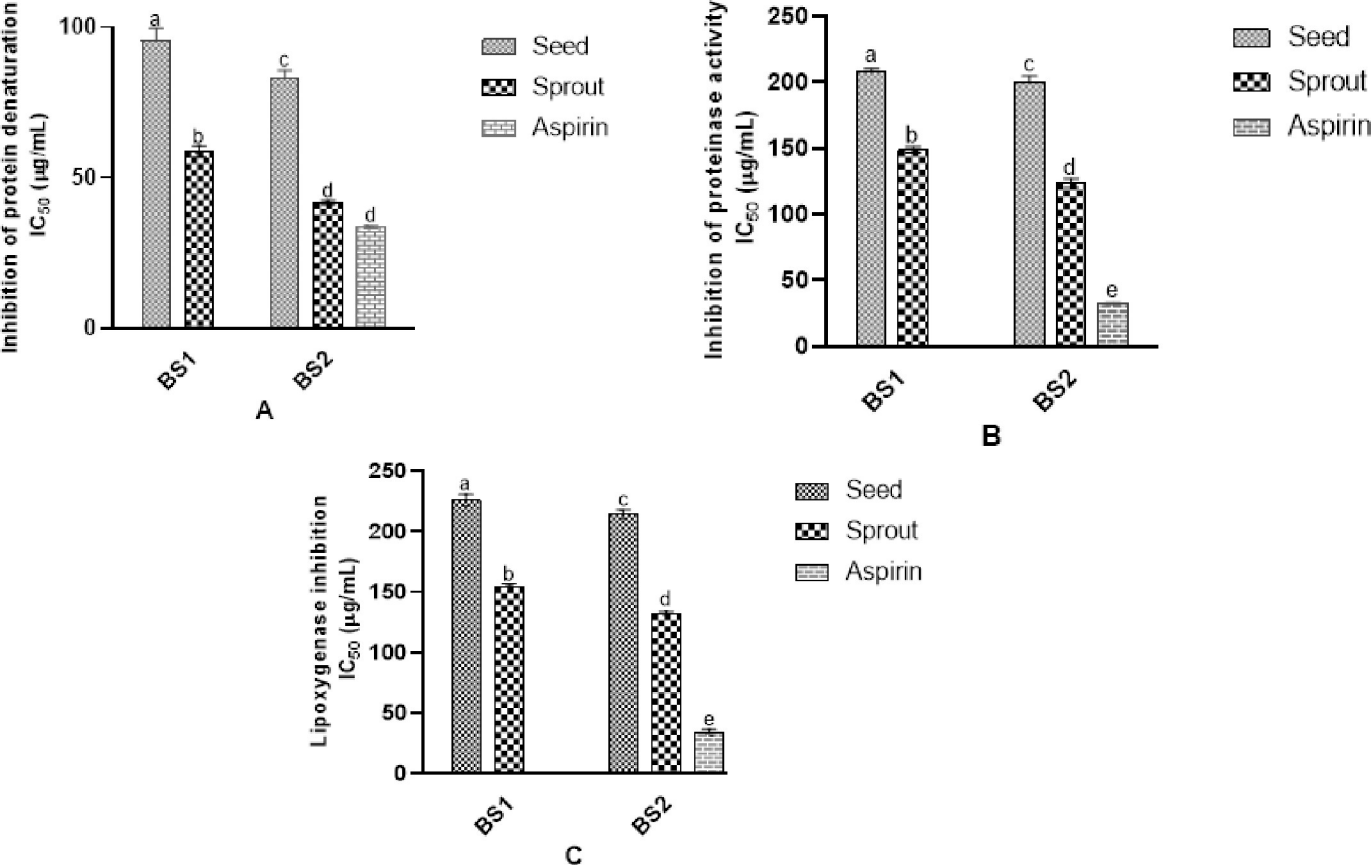
Effect of black soybean extracts (1mg/mL) to inhibit inflammatory factors. **A**) Inhibitory effects on protein denaturation IC_50_ (μg/mL), **B**) Proteinase inhibitory activity IC_50_ (μg/mL); **C**) Lipoxygenase inhibitory activity IC_50_ (μg/mL). Values represent triplicate readings and different superscripts denote significant differences (*p < 0*.*05*).

The presence of external stress or other compounds during the protein denaturation process can lead to loss of their functionality [20]. Thus, the denaturation of tissue proteins is known as a contributor to inflammation. Fig 4A is showing the effect of black soybean (varieties) seeds and sprouts against protein denaturation. The inhibitory activity against bovine albumin denaturation was found highest (IC_50_) in the sprouted extracts of BS2 (41.69±3.26 μg/mL), followed by BS1 (58.67±5.92 μg/mL) and BS2 seeds (83.18±3.52 μg/mL). The lowest activity (95.58±2.34 μg/mL) was recorded for BS1 seeds. Germination significantly protected the protein denaturation. The values of BS2 sprouts were comparable to aspirin (IC_50_ = 33.3±1.14 μg/mL).

#### 3.4.2. Proteinase inhibitory activity

Proteinases of leukocytes play role in tissue damage development during the inflammatory reactions [20]. Fig 4B represents the antiproteinase activity (IC_50_) of sample extracts. Germinated BS2 (123.72±2.96 μg/mL) showed the highest inhibitory activity, followed by BS1 sprouts (148.7±1.87 μg/mL). The values for non-germinated sample extracts were 190.27±5.73 (BS2) and 215.17±7.63 (BS1) μg/mL. A significant difference was observed in seeds and sprouts against proteinase activity. However, aspirin inhibited 32.3% proteinase at 100 μg/mL concentration.

#### 3.4.3. Lipoxygenase inhibitory activity

Lipoxygenase is the key enzyme in the biosynthesis of leukotrienes. This enzyme deoxygenizes polyunsaturated fatty acids to convert them into *cis*, *trans*-conjugated diene hydroperoxides (such as leukotrienes) that play a significant role in inflammatory diseases [18]. Fig 4C illustrate the anti-lipoxygenase activity (IC_50_) of sample extracts and the germinated seeds showed a significant enhancement (131.3±5.31 and 154.1±3.09 μg/mL in BS2 and BS1 sprouts, respectively) in the activity than non-germinated seeds. Conversely, the aspirin (100 μg/mL) values were 33.7±1.83μg/mL.

### 3.5. TPC, TFC, TAC and antioxidant activity of extracts

The result of total anthocyanin, flavonoid, phenolic contents and antioxidant capacity (DPPH, ABTS, and FRAP) of ethanol extracts were presented in Table 2. The TAC, TFC, and TPC were expressed in terms of Cyanidine 3-O-Glucoside chloride (CG3), gallic acid, and catechin equivalent, respectively. DPPH, ABTS, and FRAP assays were measured in terms of mg Trolox equiv./100 g, DW. Generally, germination enhances the phenolic contents and antioxidant activity as phenolic compounds are suggested to possess the ability to scavenge free radicals that result in the reduction of oxidative damage. Thus, this study also found that germination was effective in increasing the health-related functionalities with respect to antioxidant activities of the soybean.

**Table 2:** Total anthocyanin content (TAC), total phenolic content (TPC), total flavonoid content (TFC), and antioxidant activity (DPPH, ABTS, and FRAP) of black soybean seeds and sprouts extracts.

Samples	TAC (mg CG3 Equiv./g, DW)	TPC (mg Gallic Acid Equiv./g, DW)	TFC (mg Catechin Equiv./g, DW	FRAP (mg Trolox Equiv./g, DW)	ABTS (mg Trolox Equiv./g, DW)	DPPH (mg Trolox Equiv./g, DW)
**BS1 seed**	24.69±1.21^a^	17.51±1.47^a^	5.73±0.32^a^	11.38±2.64^a^	9.46±1.63^a^	4.29±0.87^a^
**BS1 sprout**	18.31±2.35^b^	25.92±2.82^b^	14.34±1.56^b^	20.52±2.87^b^	14.62±2.01^b^	7.28±1.29^b^
**BS2 seed**	26.89±2.87^c^	21.59±1.23^c^	8.67±0.40^c^	16.48±1.97^c^	12.39±1.27^c^	6.16±1.31^c^
**BS2 sprout**	19.36±1.92^b^	33.40±3.69^d^	20.92±3.61^d^	27.30±3.03^d^	19.02±2.18^d^	10.69±1.95^d^

All data are means ± SD of triplicate analyses. Different alphabetical letters in each column denote the level of significance (p < 0.05). BS1: black soybean-1 (Se-Um) BS2: black soybean-2 (Miryang 365), DW: dry weight.

Germination significantly reduced the TAC (measured as mg CG3 Equiv./g, DW) in both BS1 (18.31±2.3) and BS2 (19.36±1.92) that was 24.69±1.21 in BS1 seeds and 26.89±2.87 in BS2 seeds. However, TPC content significantly increased after germination and the highest increment was observed in BS2 sprouts (33.40±3.69) than seeds (21.59±1.23). Germination enhanced 8.41 mg/g content in BS1 while 11.81 mg/g content of phenolics were increased in BS2 that was calculated as mg gallic acid Equiv./g, DW. Moreover, similar trends were observed for TFC in germinated sample extracts. About 12.61 mg catechin Equiv./g, DW content was increased in BS2 sprouts and 8.61 mg catechin Equiv./g, DW in BS1 sprouts. On the other hand, the antioxidant capacity (FRAP, ABTS and DPPH) was noticed lowest in BS1 seeds and the highest values for FRAP (27.30±3.03 mg TE/g, DW), ABTS (19.02±2.18 mg TE/g, DW), and DPPH (10.69±1.95 mg TE/g, DW) assays were observed in BS2 sprouts. BS1 sprouts showed a significant increase in the FRAP (20.52±2.87 mg TE/g, DW), ABTS (14.62±2.01 mg TE/g, DW), and DPPH (7.28±1.29 mg TE/g, DW) values as compared to the seeds.

## 4. Discussion

Progressive denaturation in the function and structure of the central or peripheral nervous system characterize neurodegeneration [21]. Unlike natural products, synthetic drugs that are used against AD especially for inhibition of enzymes have some side effects, including diarrhoea, nausea, drowsiness, and headache [22]. In this study, black soybean genotypes were used to test their ability against in vitro biomarkers of AD, including oxidative stress, inflammatory factors, and cholinesterase and GABA levels were also investigated. Moreover, the alteration in the metabolites due to the germination was also studied using the UHPLC-Q-TOF-MS/MS.

In our study, several untargeted metabolites were identified, such as polyphenols, and their peak area (equal to concentration) was higher in sprouts than seeds. BS2 (Miryang 365) sprouts exhibited the highest increment of polyphenols such as gallic acid, p-coumaric acid, genistein, naringenin, daidzein (Table 2 and Fig 1A). While a higher level of (+)-catechin was identified in BS1 (Se-Um) sprouts. These findings are in accordance with Ren et al. [23], who observed an increment in the isoflavones after germination. However, cyanidin 3-glucoside was identified higher (2 folds) in the seeds than sprouts. Polyphenols can possess anti-oxidative and anti-inflammatory properties and have the potential to promote memory, learning, and cognitive functions [24]. Some essential amino acids, including L-phenylalanine, L-tryptophan, L-lysine, and L-valine, semi-essential amino acids (L-histidine and L-arginine), and non-essential amino acids (L-asparagine) were also identified. Suzuki et al. [25] stated that phenylalanine, lysine, histidine, valine, and tryptophan with other essential amino acids can improve attention, cognitive flexibility, and psychosocial functioning and can prevent cognitive decline and similar findings were observed by Sato et al. [26]. In this study, we identified those amino acids and the germination positively increased the content. Moreover, α-linolenic acid, linoleic acid, and oleic acid were also identified. The α-linolenic acid and linoleic acid may exert anti-neuro-inflammatory activity [27], and oleic acid can reduce amyloidosis [28]. Thus, metabolites identified via UHPLC-Q-TOF-MS/MS signified activities to reduce memory impairment and enhance neuroprotection. PCA (Fig 1B) analysis showed discriminated metabolite profiling of black soybean sprouts and seeds. Both seeds and sprouts were found to be divergent from each other on the basis of the abundance of identified compounds. Moreover, BS2 sprouts exhibited the highest concentration of bioactive compounds.

AChE hydrolyses ACh into acetate and choline that inhibits its neuronal functions that is known to accelerate the Aβ peptides aggregation and leads to the formation of the Aβ-AChE complex at the synaptic region of the hippocampus and contributes to neurodegeneration. Further, BChE mainly presents in the subcortical neurons and glial cells and also co-regulates cholinergic neurotransmission via hydrolysis of ACh. BChE levels increase the transformation of benign plaques to malignant plaques, resulting in the loss of neurons. Therefore, the inhibition of AChE and BChE can increase ACh levels and inhibit the Aβ-plaques formation [29]. The use of different polyphenols, either solitary or in combination, can inhibit the cholinesterase enzymes [24], and in the present study, we found that increase in polyphenols due to germination also enhanced the inhibition of enzymes. Ferhat et al. [30] stated that higher polyphenols content can exhibit strong AChE and BChE inhibitory activity, but the inhibition mechanism of both enzymes can be different. Similar results were observed in this study as higher inhibition was noticed towards BChE than AChE (Fig 2A and 2B).

Enhancement of GABA levels (Fig 3) was noted after the germination that might be due to the glutamate decarboxylase (an enzyme that catalyzes the alpha-decarboxylation of L-glutamate to form GABA). Possibly, sprouting enhanced GABA to endure stress caused by germination. Our results are comparable with Huang et al. [31], who reported a significant increase (10- fold) in the GABA content after 3^rd^ day of sprouting. A clinical study revealed that supplementation of GABA (200 mg/ day) for 12 weeks maintained or improved the cognitive function, working memory, and mental health of adults subjects aged over 40 years. Thus, germination of black soybean can be a valuable source to obtain GABA to potentially treat neurological disorders [32].

In the present study, the in vitro ability of raw and sprouted black soybeans to inhibit the inflammation factors (protein denaturation, proteinase, and lipoxygenase: essential mediators in inflammatory events [18, 20, 33]) has been tested. Results showed that germination effectively enhances the ability of black soybeans to inhibit protein denaturation, proteinase, and lipoxygenase. In this study, BS2 sprouts exhibited the highest inhibitory activity that might be due to the higher content of bioactive compounds and the antioxidant properties. Truong et al. [20] stated that anti-inflammatory activities of foods might be due to the higher phenolic and flavonoid content. Contrary, Eum et al. [11] claimed that bioactive factors such as antioxidant activity, TFC, and TPC do not necessarily reflect the anti-inflammatory properties but the aglycones (such as genistein and daidzein) are more effective. However, we found higher bioactive factors, genistein, daidzein, naringenin, p-coumaric acid, and (+)-catechin (Table 1 and heat map) in BS2 sprouts that showed the highest inhibition of inflammatory factors.

Furthermore, polyphenols (TPC, TFC, and TAC) were also examined. An increase in phenolic and flavonoid content was noticed during germination and these values are in accordance with Min et al. [34], who also observed an increase in TPC and TFC content after germination. Further, a decrease in TAC was observed in sprouts and similar findings were observed by Min et al. [34]. However, Sutharut et al [35] found an increase in TAC after germination of colored rice. In our prospect, a decrease in anthocyanin content could be due to the hydration or loss of water molecules or activation of the enzyme (anthocyanase or polyphenoloxidase) during soaking and germination.

Plant-based diets are rich in antioxidant phytochemicals and can reduce the pathogenesis and progression of AD that is associated with oxidative stress. Moreover, oxidative stress can also lead to selective loss of cholinergic neurons in the brain and promote amyloid protein deposition and neurotoxicity in the brain [22]. Results have shown that germination of black soybean significantly increased the antioxidant capacity (DPPH, ABTS, and FRAP). An increase in TPC and TFC (gallic acid, naringenin (+)-catechin, genistein, and daizein) increased the antioxidant capacity [36]. Thus, germination of black soybean varieties can effectively inhibit oxidative stress, inflammatory factors, cholinesterase enzymes and can increase the GABA content.

## Conclusion

The current study explored that new genotypes of black soybean (Miryang 365 and Se-Um) have antioxidative, inhibition of inflammatory factors and anticholinesterase potential and germination enhances these properties along with GABA content. Moreover, present findings reveal a promising potential of using germination to alter the metabolites for developing healthy functional seeds and foods to prevent/inhibit neurodegenerative diseases. Additionally, further study is required in animal models to substantiate the in vitro findings.

## Supporting information

S1 Fig Black soybean varieties and their germinated seeds. (PDF)

S1 Table Initial screening data (%) of the raw and germinated black soybean over 5 days (ethanol extracts: 1mg/mL) samples. (PDF)
