# Supplementary figures and images for: Comprehensive profiling of bioactive compounds in germinated black soybeans via UHPLC-ESI-QTOF-MS/MS and their anti-Alzheimer’s activity
Source: PLoS One. 2022 Jan 28;17(1):e0263274. doi: 10.1371/journal.pone.0263274 (PMC8797171; doi:10.1371/journal.pone.0263274)

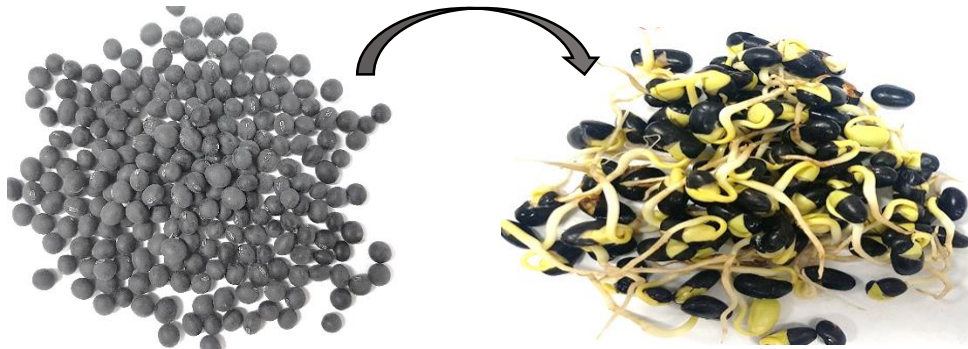

세움 (Se-Um: BS1)

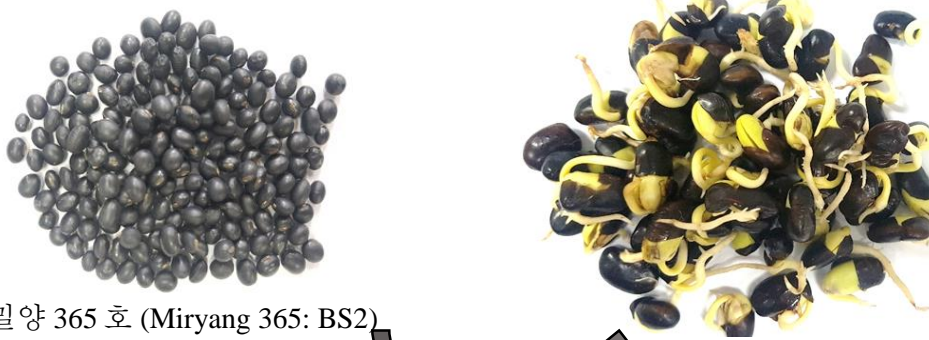

밀양 365 호 (Miryang 365: BS2)

S1 Fig: Black soybean varieties and their germinated seeds

Supplement: S1 Fig — (PDF) [file pone.0263274.s001.pdf]
