# Supplementary material for: Comprehensive profiling of bioactive compounds in germinated black soybeans via UHPLC-ESI-QTOF-MS/MS and their anti-Alzheimer’s activity
Source: PLoS One. 2022 Jan 28;17(1):e0263274. doi: 10.1371/journal.pone.0263274 (PMC8797171; doi:10.1371/journal.pone.0263274)
Supplement: S1 Table — (PDF) [file pone.0263274.s002.pdf]

S1 Table: Initial screening data (%) of the raw and germinated black soybean over 5 days (ethanol extracts: 1mg/mL) samples.

a)

**Germination days**	**DPPH**	**ABTS**	**FRAP**	**Inhibition of protein denaturation**	**Inhibition of proteinase**	**Inhibition of lipoxygenase**
0	41.03±1.82[a]	54.89±1.21[a]	53.06±1.49[a]	42.76±2.93[a]	33.01±2.45[a]	30.42±0.59 [a]
1	54.16±1.63[b]	61.32±2.98[b]	62.56±2.03[b]	56.92±1.83[b]	40.42±2.21[b]	37.51±1.01[b]
2	63.40±0.71[c]	72.53±2.30[c]	74.61±1.83[c]	68.03±1.59[c]	47.94±1.92[c]	44.09±2.37[c]
3	81.28±0.60[d]	84.06±0.91[d]	88.43±1.01[d]	82.23±2.47[d]	63.30±3.03[d]	58.44±1.45[d]
4	76.47±1.02[e]	80.59±1.02[de]	84.91±1.45[de]	76.52±2.15[e]	61.92±2.69[de]	55.68±2.41[de]
5	74.62±0.59[e]	76.09±0.99[e]	79.23±2.41[e]	73.43±1.72[e]	58.15±0.52[e]	51.75±1.64[e]
Standard (Trolox/Aspirin)	90.48±2.71	92.10±1.41	91.05±3.06	89.14±3.47	91.54±2.78	90.81±1.52

b)

**Germination days**	**DPPH**	**ABTS**	**FRAP**	**Inhibition of protein denaturation**	**Inhibition of proteinase**	**Inhibition of lipoxygenase**
0	43.51±2.04[a]	54.02±0.43[a]	53.20±0.56[a]	44.59±2.61[a]	34.52±1.80[a]	31.58±1.68[a]
1	57.52±1.88[b]	63.79±0.98[b]	61.36±1.20[b]	58.02±1.98[b]	44.09±0.67[b]	39.09±1.57[b]
2	69.02±2.48[c]	75.64±1.75[c]	79.66±1.32[c]	70.67±1.43[c]	53.84±1.65[c]	52.82±3.44[c]
3	85.60±1.51[d]	88.81±2.09[d]	92.49±1.76[d]	86.91±1.57[d]	68.92±1.08[d]	61.53±2.40[d]
4	79.33±1.73[e]	81.30±1.38[e]	88.34±1.84[de]	79.09±0.79[e]	64.01±0.96[de]	54.92±2.17[e]
5	76.75±2.14[e]	79.23±1.05[e]	83.91±0.81[e]	76.31±1.60[e]	58.42±1.29[e]	49.98±1.94[e]
Standard (Trolox/Aspirin)	90.52±3.69	93.01±1.99	92±2.54	88.93±1.55	91.67±2.41	89.41±2.05

Table a shows data for BS1 (Se-Um) and Table for BS2 (Miryang 365) in percentage (%). Results are expressed as the mean ± SD of triplicate analyses and different superscripts denote significant differences ($p < 0.05$) in each column. Trolox used as a standard for DPPH, ABTS, and FRAR, while Aspirin for inhibition of protein denaturation, inhibition of proteinase, and lipoxygenase.
